# Effects of Printing Layer Thickness and Thermocycling on the Mechanical, Morphological, Optical, and Dimensional Properties of Directly Printed Clear Aligners

**DOI:** 10.3390/polym18182281

**Published:** 2026-09-18

**Authors:** Isra Dilshad Rostum, Omar Sabah Mohammed Jamil Ali

**Affiliations:** 1 Department of Preventive, Orthodontics, and Pediatric Dentistry, College of Dentistry, University of Duhok, Duhok 42001, Kurdistan Region, Iraq; 2 Department of Orthodontics, Dentistry Faculty, Tishk International University, Erbil 44001, Kurdistan Region, Iraq; 3 Department of Preventive, Orthodontics, and Pediatric Dentistry, College of Dentistry, University of Mosul, Mosul 41001, Iraq

**Keywords:** orthodontic appliance, LCD stereolithography, layer thickness, thermocycling, Graphy Tera Harz TA-28, flexural properties, tensile strength, Vickers microhardness, light transmittance, field-emission scanning electron microscopy, dimensional accuracy

## Abstract

Directly printed clear aligners may streamline orthodontic workflows but must withstand intraoral aging. This in vitro study evaluated the effects of layer thickness (50, 100, and 150 µm) and thermocycling on mechanical, morphological, optical, and dimensional properties of Graphy Tera Harz TA-28 material. Specimens underwent standardized centrifugation and nitrogen post-curing, followed by tensile and flexural testing, Vickers microhardness, normalized light transmittance, dimensional analysis, and field-emission scanning electron microscopy (FESEM). Thermocycling comprised 250 cycles between 5 and 55 °C. Layer thickness significantly affected strain at break, flexural force, bending strength, bending modulus, microhardness, and normalized light transmittance. The 50 µm group showed the highest strain at break and greater flexural performance than the 100 and 150 µm groups. Thermocycling reduced Young’s modulus and flexural outcomes. Significant layer thickness × thermocycling interactions occurred for stress at peak, deflection at peak, microhardness, normalized light transmittance, trueness (*p* = 0.008), and precision (*p* = 0.034). FESEM showed visible layer boundaries and surface heterogeneity with thicker layers, particularly in thermocycled 150 µm specimens. Overall, 50 µm provided favorable mechanical and dimensional performance after thermocycling, but produced the greatest reduction in light transmittance. Selection requires balancing mechanical and dimensional performance against optical stability after aging.

## 1. Introduction

Clear aligner therapy provides an aesthetic alternative to fixed orthodontic appliances. Its clinical effectiveness depends on the accuracy and predictability of planned tooth movement, the forces delivered by the appliance, and the retention of material properties during intraoral service. A systematic review found low-to-moderate certainty of evidence for some tooth movements and insufficient evidence for others [1]. These limitations highlight the need to investigate not only treatment protocols but also the materials and manufacturing workflows used to produce aligners.

Conventional clear aligners are manufactured by thermoforming a thermoplastic sheet over a dental model. Thermoforming can alter material properties, while intraoral exposure may further affect aligner behavior during treatment [2]. Direct three-dimensional (3D) printing offers an alternative digital workflow in which an aligner is manufactured directly from its digital design, without an intermediate physical model. This approach may provide greater control over aligner geometry and thickness; however, the technical and clinical evidence base for directly printed aligners remains comparatively limited [2].

Dimensional accuracy is particularly relevant for direct-printed aligners because deviations in the intaglio surface may affect seating and, consequently, the transfer of the planned appliance geometry to the dentition. Previous in vitro studies have evaluated trueness by superimposing scanned aligner meshes on a reference STL design and precision by comparing repeated aligners within a group [3,4]. Koenig et al. [3] reported greater trueness and precision for one direct-printed aligner material than for two thermoformed materials; however, this result was obtained using a different resin and fabrication workflow and cannot be generalized to Graphy Tera Harz TA-28. Similarly, artificial-saliva aging has been evaluated as a determinant of aligner trueness and precision, indicating that dimensional outcomes should be considered together with material type and aging conditions [4].

In LCD-based stereolithography, printing layer thickness is a selectable process parameter that may influence polymerization uniformity, interlayer continuity, surface reproduction, and the mechanical behavior of the printed appliance. Thinner layers require more printing cycles and may increase production time, whereas thicker layers require fewer cycles and may shorten fabrication time. The magnitude and direction of these effects cannot be inferred from layer height alone because they depend on the resin, printer, build orientation, exposure settings, and post-processing protocol.

Previous studies of DLP dental resins illustrate this workflow dependence. Farkas et al. reported that printing orientation and layer thickness affected tensile properties, with the highest tensile values at 50 µm [5]. Moreover, Alshamrani et al. found the highest flexural strength at 100 µm in another printed dental resin and showed that post-printing conditions affected flexural strength and Vickers hardness [6]. Together, these findings indicate that the effect of printing layer thickness is material- and workflow-dependent. The comparison of 50, 100, and 150 µm is, therefore, relevant to direct-print aligner manufacturing because it evaluates fine, intermediate, and coarser layer settings within one controlled TA-28 workflow.

Layer thickness was selected as the manufacturing variable because it is a practical slicing choice that directly changes the number of interfaces through the specimen thickness and can be implemented within a controlled resin–printer workflow. Other process parameters can also be consequential. In particular, exposure time per layer determines the delivered light dose and can alter degree of conversion and thermo-mechanical behavior; in polyurethane-based aligner resins, UV-exposure duration affected degree of conversion, glass-transition temperature, and mechanical response [7]. Build orientation changes the relationship between the loading direction and layer interfaces. Published direct-aligner studies report orientation-dependent mechanical, roughness, and color-stability outcomes [8], although another study found no significant horizontal-versus-vertical orientation difference under its tested conditions [9]. These findings support treating layer thickness, exposure, orientation, and post-processing as interacting workflow parameters rather than assuming a universal effect of any single setting.

The present experiment was intentionally limited to layer-thickness workflow and thermocycling in a 3 × 2 factorial design. Exposure, build orientation, printer, resin, washing, nitrogen post-curing, and final thermal conditioning were held constant within the manufacturer-recommended workflow (not for 150 µm; see Appendix A) except for the layer-thickness-specific settings reported in Table A1. This design does not estimate the independent effects of exposure time or orientation, nor their interactions with aging; rather, it isolates the performance of three practically achievable layer-thickness-specific TA-28 workflows under one standardized manufacturing and post-processing protocol.

Graphy Tera Harz TA-28 is a light-curable resin developed for directly printed orthodontic aligners and intended for intraoral use after standardized post-processing. Bleilöb et al. reported that, under the manufacturer-recommended 20-min post-curing protocol, cell viability declined as specimen thickness increased but remained below the EN ISO 10993-5 cytotoxicity threshold [10]. Post-processing was, therefore, standardized in the present study to isolate the effects of the printing workflow.

TA-28 was selected because it is a biocompatible and commercially and locally available resin intended for direct printing of clear aligners and has been evaluated in previous direct-aligner studies [10,11,12,13]. The present study was designed as a controlled in vitro evaluation of the effects of printing layer thickness and thermocycling within one resin–printer–post-processing workflow. Accordingly, the findings should be interpreted as workflow-specific rather than generalized to other resin–printer systems.

Post-processing conditions provide further justification for this controlled approach. Rinsing protocol and polymerization environment affect the flexural modulus and hardness of TC-85 polyurethane aligners; in one study, centrifugation followed by nitrogen polymerization produced the highest flexural modulus [14]. Mattle et al. reported no significant difference in the mechanical effects of nitrogen-atmosphere and heat post-curing, indicating that post-processing effects are protocol-dependent [15]. Standardization of the post-processing workflow is, therefore, necessary to avoid falsely attributing curing-related effects to layer thickness.

Thermal aging is also relevant because aligners experience repeated temperature changes during intraoral use that may affect the mechanical and optical behavior of photocurable polymers. Can et al. compared unused Graphy TC-85DAC aligners with aligners retrieved after one week of clinical wear and found no significant differences in the indentation-based mechanical properties measured [16]. However, the material, aging conditions, and measured outcomes differed from those of the present study, and printing layer thickness was not evaluated.

Therefore, this in vitro study investigated the effects of printing layer thickness (50, 100, and 150 µm) and thermocycling on the tensile and flexural properties, Vickers microhardness, normalized light transmittance, and dimensional accuracy of directly printed Graphy Tera Harz TA-28 aligner material. Field-emission scanning electron microscopy (FESEM) was used to qualitatively evaluate surface and tensile-fracture morphology. The null hypotheses were that printing layer thickness, thermocycling, and their interaction would not affect the measured mechanical, optical, or dimensional outcomes.

## 2. Materials and Methods

### 2.1. Study Design and Experimental Groups

An in vitro controlled factorial design experiment (3 × 2) was used to evaluate the effect of different printing layer thicknesses (50, 100, and 150 µm) and thermocycling (thermocycled vs. non-thermocycled) on the directly printed orthodontic aligner resin. The experimental groups are presented in Table 1. Samples were manufactured and post-processed under standardized conditions and tested for tensile properties, flexural properties, Vickers microhardness, surface characteristics, optical transmittance, and dimensional accuracy.

### 2.2. Materials and Sample Preparations

A commercially available 3D-printable clear-aligner resin (Tera Harz TA-28; Graphy Inc., Seoul, Republic of Korea), described as a urethane-methacrylate-based shape-memory photopolymer [11], was used in this study. The manufacturer does not disclose the complete chemical composition or a single molecular formula of this proprietary resin. Specimens were fabricated using an LCD-based stereolithography 3D printer (Uniz NBEE, UNIZ Technology LLC, San Diego, CA, USA) from STL files that are generated using CAD software (Autodesk 3ds Max 2025; Autodesk Inc., San Francisco, CA, USA).

Specimen geometries were selected according to the test method used. Tensile specimens conformed to ASTM D638 Type V [17], and flexural specimens conformed to ISO 20795-2 [18]. Rectangular specimens with dimensions 50 mm × 10 mm × 0.6 mm were prepared for Vickers microhardness testing using the procedure of Atta et al. [19]. Optical specimens were prepared with dimensions of 8 mm (length), 50 mm (width) and 0.5 mm (thickness) [20].

The STL files were imported into the manufacturer’s slicing software, and the specimens were printed with layer thicknesses of 50, 100, or 150 µm. The 50 and 100 µm groups used the manufacturer’s recommended print settings. No manufacturer-recommended printing conditions exist for the 150-µm condition; thus, the exposure time, LED power, lifting height, and lifting speed for the 150-µm condition were experimentally determined through preliminary printing trials. Specimens were printed with conditions so that no incomplete curing, delamination, or deformation was observed. The final printing conditions used for each group are provided in Table A1. The fabrication parameters were kept constant, except for those shown in Table A1.

For the flat specimens used in mechanical and optical testing, the build orientation was maintained at 0° relative to the build platform, and no supports were used. Full-arch aligners fabricated for dimensional-accuracy assessment were printed at 45° relative to the build platform using distributed supports, with reduced support density on the labial surface. Within each specimen type, build orientation and support configuration were kept constant across the 50, 100, and 150 µm layer-thickness workflows. Exposure and movement settings were fixed within each workflow as reported in Table A1; thus, the study compares layer-thickness-specific manufacturing workflows rather than layer thickness as an isolated geometric variable.

After printing, specimens were removed from the build platform. Uncured resin on the surface of specimens was removed by centrifugation using a Tera Harz Spinner (THSP; Graphy Inc., Seoul, Republic of Korea) at 500 rpm for 6 min. Post-curing of the specimens was conducted in a nitrogen atmosphere in a Tera Harz Cure N2 chamber (THC-2; Graphy Inc., Seoul, Republic of Korea) for 20 min (10 min per side). Following post-curing, all specimens were immersed in boiling water (approximately 100 °C) for 2 min as a standardized final thermal-conditioning step before subsequent testing, following the post-processing procedure reported for directly printed Tera Harz TA-28 specimens by Bleilöb et al. [10]. Defective specimens (post-cured specimens having distortion, incomplete curing, or any surface irregularity or structural damage) were excluded from testing. In the quantitative analysis, each group was represented by five specimens fabricated independently, unless otherwise stated. One specimen from each experimental group was analyzed by FESEM to characterize the pre-fracture surface morphology and post-fracture cross-sectional features.

### 2.3. Thermocycling Procedure

In each printing layer thickness group, half of the samples were thermocycled and half were not. A modified protocol based on Alweneen et al. [21] was used. Accordingly, 250 cycles were used in the present study to approximate 7 days of aligner wear, corresponding to the wear protocol reported for directly printed Graphy aligners [22]. The specimens were thermocycled in water at 5 and 55 °C, respectively. A dwell time of 15 s was maintained. A commercial thermocycler (SD Mechatronik GmbH, Feldkirchen-Westerham, Germany) was used. After thermocycling, specimens were gently dried and stored until testing.

### 2.4. Mechanical Testing

#### 2.4.1. Tensile Test

Tensile testing was performed using ASTM D638-V [17] dumbbell specimens (length = 63.5 mm, width = 9.53 mm, thickness = 2 mm, and gauge width = 3.18 mm; *n* = 5 per group) on a universal testing machine (Cussons P-Series, Salford, UK). Specimens were placed between an upper and lower grip and aligned with the direction of loading. A uniaxial tensile load was applied at a crosshead speed of 1 mm/min until fracture. Load-displacement data were acquired using the winTest Analysis EC software (Testometric Co. Ltd., Rochdale, UK) and converted to stress-strain curves. Stress at peak, strain at break, and Young’s modulus in the linear-elastic region were recorded.

#### 2.4.2. Three-Point Flexural Testing

Rectangular specimens were prepared according to ISO 20795-2 [18] (64 mm × 10 mm × 3.3 mm; *n* = 5 per group) for the three-point flexural tests. The specimens were tested in the same universal testing machine (Cussons P-Series, Salford, UK), on two supports with a support span of 50 mm. Loading comprised a centered pin loading, applied at a constant crosshead speed of 5 mm/min until failure, and load-deflection data were recorded using the winTest Analysis EC software. Force at peak, deflection at peak, flexural strength, and flexural modulus were calculated.

#### 2.4.3. Vickers Microhardness Testing

Vickers microhardness test was performed using a digital microhardness tester (DHV-1000; Shanghai Precision Instruments Co., Ltd., Shanghai, China). Rectangular specimens (50 mm × 10 mm × 0.6 mm; *n* = 5 per group) were fabricated according to the method described by Atta et al. [19]. The test was performed with a diamond pyramidal indenter, a load of 1.96 N (200 gf), and a dwell time of 15 s. Three indentations were made on each sample with sufficient separation in different places on the flat and smooth surface to prevent overlapping of the deformation zones. The hardness was evaluated by measuring the diagonal lengths of the indentation using a microscope integrated into the instrument and automatically calculating the Vickers hardness (HV) from the software of the device. The mean of the three readings was used as the specimen-level value for analysis.

### 2.5. Field-Emission Scanning Electron Microscopy Analysis

The surface morphology and fracture characteristics of the specimens were analyzed using FESEM (FEI F50; FEI Company, Hillsboro, OR, USA) operated at an accelerating voltage of 30 kV under high-vacuum conditions. The specimens were mounted on aluminum stubs using conductive carbon tape. Since the resin is an electrical insulator, any charge buildup was avoided by sputter-coating a thin layer of gold onto the samples. Representative images were photographed at different magnifications to provide qualitative comparisons. The outer faces pre-fracture were tested for their printing morphology and layer patterns. After tensile testing, the fracture surfaces were imaged to qualitatively assess fracture patterns and features consistent with interlayer failure.

### 2.6. Optical Properties

The light transmittance of the samples was measured by a UV-Visible spectrophotometer (BK-D560; BIOBASE, Jinan, China). The rectangular specimens (8 × 50 × 0.5 mm; *n* = 5 per group) were placed in the middle of the sample holder for each measurement, and the device was calibrated in accordance with the manufacturer’s specifications before testing. Following cleaning, three readings were recorded and averaged as the light transmittance over the wavelength range of 350–750 nm.

For comparative analysis, normalized light transmittance was quantified as the percentage area under the transmittance curve (AUC), calculated using LabPlot (Version 2.12.1; LabPlot Development Team, KDE Community, Berlin, Germany). The measured AUC was normalized to the theoretical maximum corresponding to 100% transmittance over the wavelength range of 350–750 nm (400 nm range). Transparency was calculated using the following equation:

$$\mathrm{Transparency}\left(\%\right)=\frac{\mathrm{AUC}}{100\times (750-350)}\times 100$$

The calculated transparency (%) obtained from the normalized AUC was used as the optical outcome variable to evaluate the effects of printing layer thickness and thermocycling.

### 2.7. Dimensional Accuracy Assessment

Full-arch aligners were fabricated from a maxillary reference STL obtained from an intraoral scan of an adult retrieved from the orthodontic department archive. The digital model was used solely as the reference geometry for aligner fabrication and dimensional analysis. Aligners were designed with a uniform nominal thickness of 0.50 mm and were printed using the 50, 100, and 150 µm layer-thickness workflows. For each workflow, five aligners were assigned to the non-thermocycled condition and five to the thermocycled condition.

Dimensional accuracy was evaluated as trueness and precision using digital scanning and three-dimensional mesh superimposition. Trueness was defined as the agreement between the scanned intaglio surface of an individual fabricated aligner and the corresponding reference STL. Precision was defined as the reproducibility of aligner geometry among independently fabricated specimens within the same experimental condition. This approach is consistent with previous aligner studies that assessed trueness by comparison with the reference STL and precision by comparison among specimens produced under the same condition [3].

Before scanning, the aligners were immersed in water maintained at 37 °C for 5 min as a standardized thermal-conditioning step to initiate shape recovery. Each aligner was then immediately seated on its corresponding dental cast and retained until visually stable. This procedure standardized the pre-scanning condition of the shape-memory aligners and was not intended to simulate intraoral aging. Specimens were then dried with an air stream. A thin, uniform layer of Stone scanning spray (DENTACO GmbH & Co. KG, Essen, Germany) was applied to reduce reflection, and excess spray was removed with air. Each aligner was stabilized on a contrasting background with sticky wax and scanned with an intraoral scanner (TRIOS 3; 3Shape A/S, Copenhagen, Denmark). The scan files were exported in STL format and imported into Geomagic Control X (version 2022.1; 3D Systems, Rock Hill, SC, USA).

For trueness, the STL file of each scanned aligner was compared with the original reference STL. Following initial alignment, best-fit alignment was performed with the analysis restricted to the intaglio surface; islands and isolated regions not belonging to the aligner were excluded. The root-mean-square (RMS) surface deviation, expressed in millimeters (mm), was recorded for each comparison, with lower RMS values indicating greater dimensional agreement. RMS has been used as an overall measure of trueness in direct-printed aligner comparisons [3].

For precision, the five independently fabricated aligners within each experimental condition were compared pairwise using the same initial-alignment, best-fit-alignment, and three-dimensional-comparison workflow. All unique pairwise combinations were evaluated, resulting in 10 comparisons per experimental condition (1–2, 1–3, 1–4, 1–5, 2–3, 2–4, 2–5, 3–4, 3–5, and 4–5). The root-mean-square (RMS) surface deviation, expressed in millimeters (mm), was recorded for each comparison, with lower RMS values indicating greater manufacturing reproducibility. The color-map tolerance was set to ±0.10 mm, and the displayed deviation range was −1.00 to +1.00 mm.

### 2.8. Statistical Analysis

Statistical analysis was performed using Minitab (version 21.4.2, Minitab LLC, State College, PA, USA). For each outcome, descriptive statistics are reported as mean ± standard deviation. Data normality and homogeneity of variance were tested using the Anderson–Darling and Levene’s tests, respectively.

The effect of printing layer thickness (50, 100, and 150 µm) and thermocycling condition (non-thermocycled and thermocycled), as well as their interaction, was evaluated using two-way analysis of variance (ANOVA). Tensile, flexural, and normalized light-transmittance outcomes were analyzed on their original scales after the model assumptions were satisfied. Vickers microhardness data were subjected to Box–Cox transformation before two-way ANOVA because the original-scale residuals did not satisfy the normality assumption. For dimensional-accuracy outcomes, transformed trueness and precision responses were used for inferential analysis after confirming residual normality and homogeneity of variance. Descriptive trueness and precision values are reported on the original scale in millimeters (mm).

When a statistically significant main effect or interaction was identified, Tukey’s honestly significant difference (HSD) test was used for post hoc pairwise comparisons as appropriate. Statistical significance was set at *p* < 0.05.

## 3. Results

### 3.1. Mechanical Properties

#### 3.1.1. Tensile Properties

**Figure 1:**
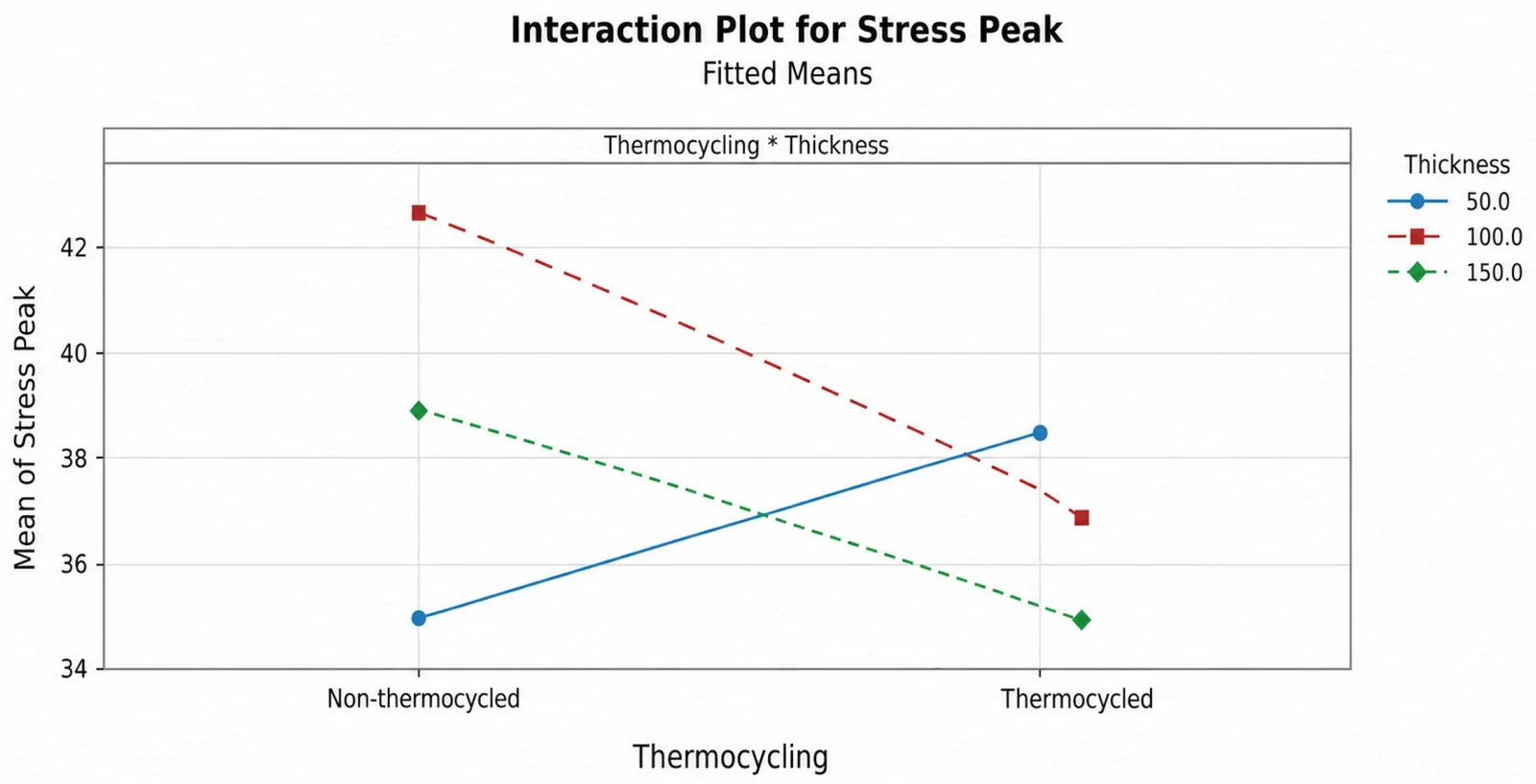
Interaction plot showing the effects of thermocycling and printing layer thickness on stress at peak.

Tensile-property descriptive statistics are presented in Table 2, and the two-way ANOVA results are summarized in Table 3. For stress at peak, the thermocycling × printing layer thickness interaction was statistically significant (*p* = 0.035), whereas the main effects of thermocycling (*p* = 0.159) and printing layer thickness (*p* = 0.179) were not significant. Because the interaction was significant, the effects of thermocycling and printing layer thickness were interpreted jointly (Figure 1). Stress at peak increased after thermocycling in the 50 µm group but decreased in the 100 and 150 µm groups.

For strain at break, printing layer thickness had a statistically significant effect (*p* = 0.003), whereas the main effect of thermocycling (*p* = 0.858) and the thermocycling × printing layer thickness interaction (*p* = 0.204) were not significant. Descriptively, the 50 µm group exhibited the highest strain-at-break values under both thermocycling conditions. Thermocycling increased strain at break in the 50 µm group, produced no change in the group mean in the 100 µm group, and decreased it in the 150 µm group. Young’s modulus was significantly affected by thermocycling (*p* = 0.001), while printing layer thickness (*p* = 0.172) and the thermocycling × printing layer thickness interaction (*p* = 0.451) were not significant. Overall, thermocycling reduced Young’s modulus across the three printing layer thicknesses.

#### 3.1.2. Three-Point Flexural Properties

Flexural-property descriptive statistics are shown in Table 4 and two-way ANOVA results are presented in Table 5. There was a statistically significant decrease in force at peak, flexural strength and flexural modulus after thermocycling (*p* < 0.001). Printing layer thickness also had a significant effect on these properties (all *p* < 0.001), with the 50 µm group having significantly higher values than the 100 and 150 µm groups (Tukey HSD, *p* < 0.05), between which no significant difference was observed (Tukey HSD, *p* > 0.05).

**Figure 2:**
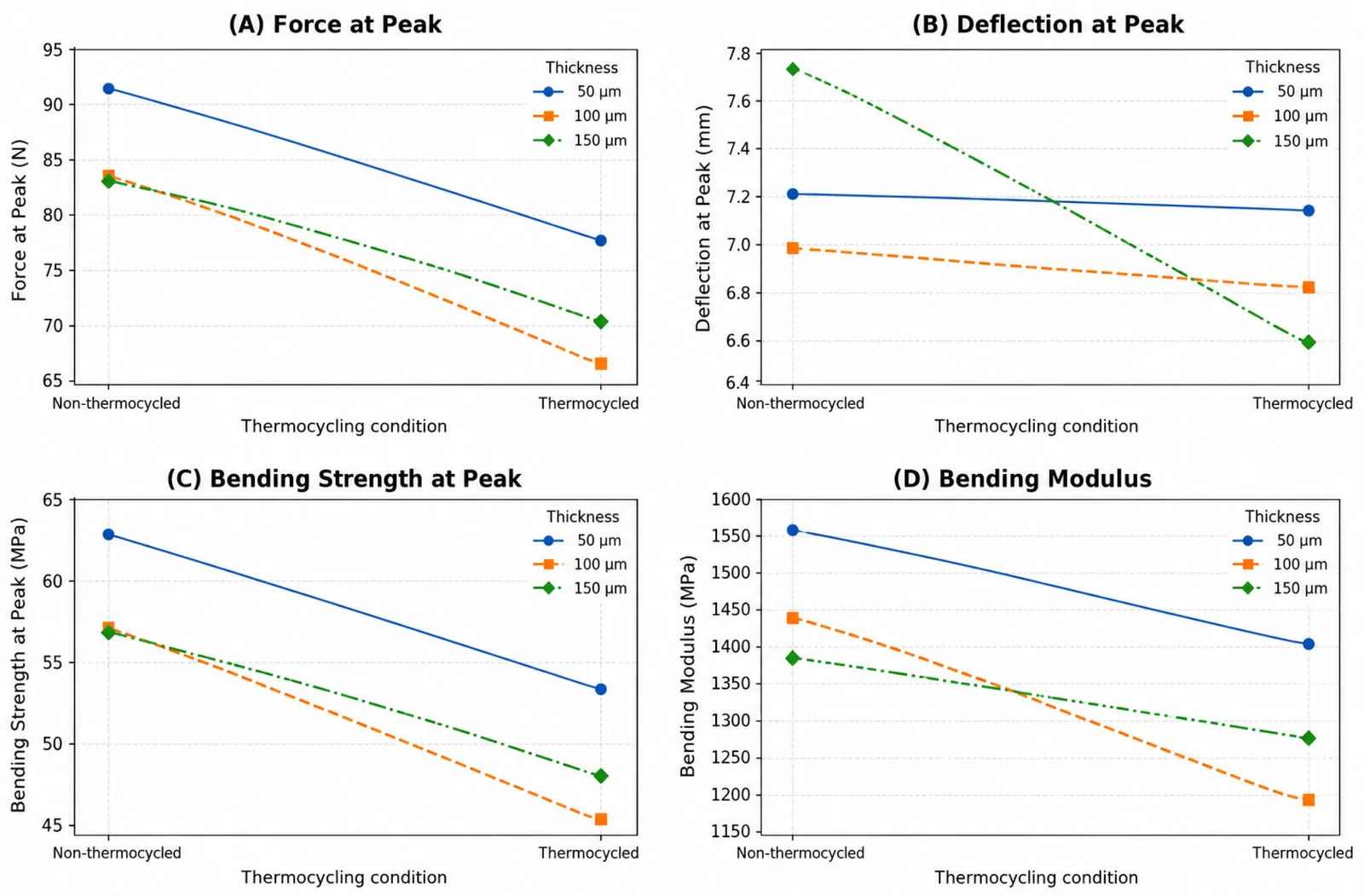
Interaction plots showing the effects of printing layer thickness and thermocycling on (**A**) force at peak, (**B**) deflection at peak, (**C**) bending strength, and (**D**) bending modulus of directly printed aligner specimens.

Thermocycling had a significant effect on peak deflection (*p* = 0.005), but printing layer thickness did not (*p* = 0.193). The thermocycling × printing-layer thickness interaction effect was significant only for peak deflection (*p* = 0.010), with the greatest peak deflection decrease seen in the 150 µm group (Figure 2B). No significant thermocycling × layer-thickness interaction was observed for force at peak, flexural strength, or flexural modulus (*p* > 0.05).

#### 3.1.3. Vickers Microhardness Findings

The Vickers microhardness means and standard deviations of each group are shown in Table 6. The greatest mean hardness value was for the non-thermocycled 150 µm group, whereas the lowest mean value was observed in the 100 µm group under thermocycled conditions.

Two-way ANOVA results showed that printing layer thickness and the layer-thickness × thermocycling interaction were significant (*p* < 0.05), while the main effect of thermocycling alone was not significant (*p* = 0.055) (Table 7).

**Figure 3:**
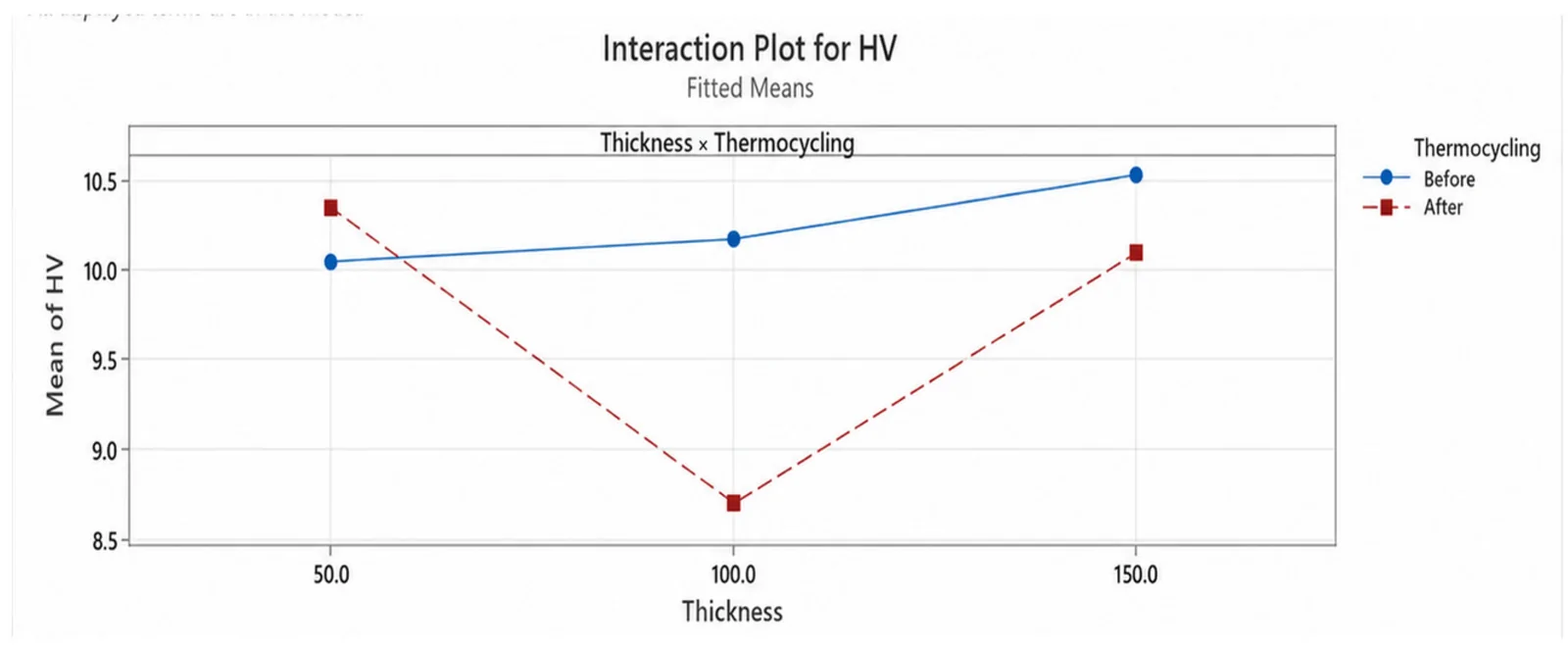
Interaction plot showing the effects of printing layer thickness and thermocycling on Vickers microhardness. Thermocycling produced a small numerical increase at 50 µm and reductions at 100 and 150 µm, with the greatest decrease observed at 100 µm. The non-parallel lines illustrate the significant printing layer thickness × thermocycling interaction (*p* = 0.037).

The effect of thermocycling on Vickers microhardness varied non-linearly with printing layer thickness. A small numerical increase was observed in the 50 µm group, whereas reductions were observed in the 100 and 150 µm groups, with the greatest decrease occurring at 100 µm. This variation was consistent with the significant printing layer thickness × thermocycling interaction (*p* = 0.037; Figure 3).

### 3.2. Field-Emission Scanning Electron Microscopy Findings

**Figure 4:**
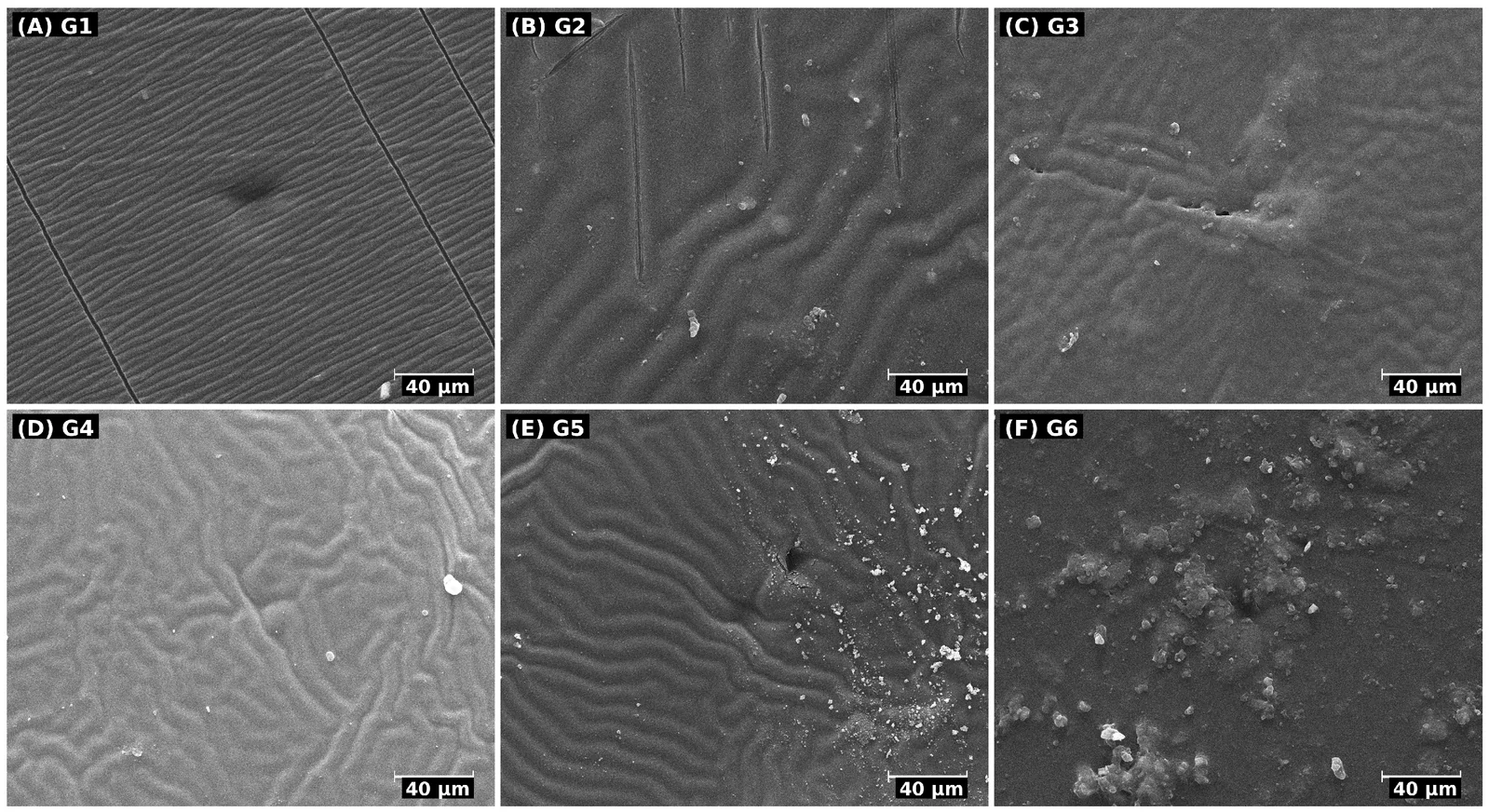
Representative pre-fracture FESEM micrographs of directly printed aligner specimens at 2000× magnification according to printing layer thickness and thermocycling condition: (**A**) G1, 50 µm non-thermocycled; (**B**) G2, 50 µm thermocycled; (**C**) G3, 100 µm non-thermocycled; (**D**) G4, 100 µm thermocycled; (**E**) G5, 150 µm non-thermocycled and (**F**) G6, 150 µm thermocycled.

**Figure 5:**
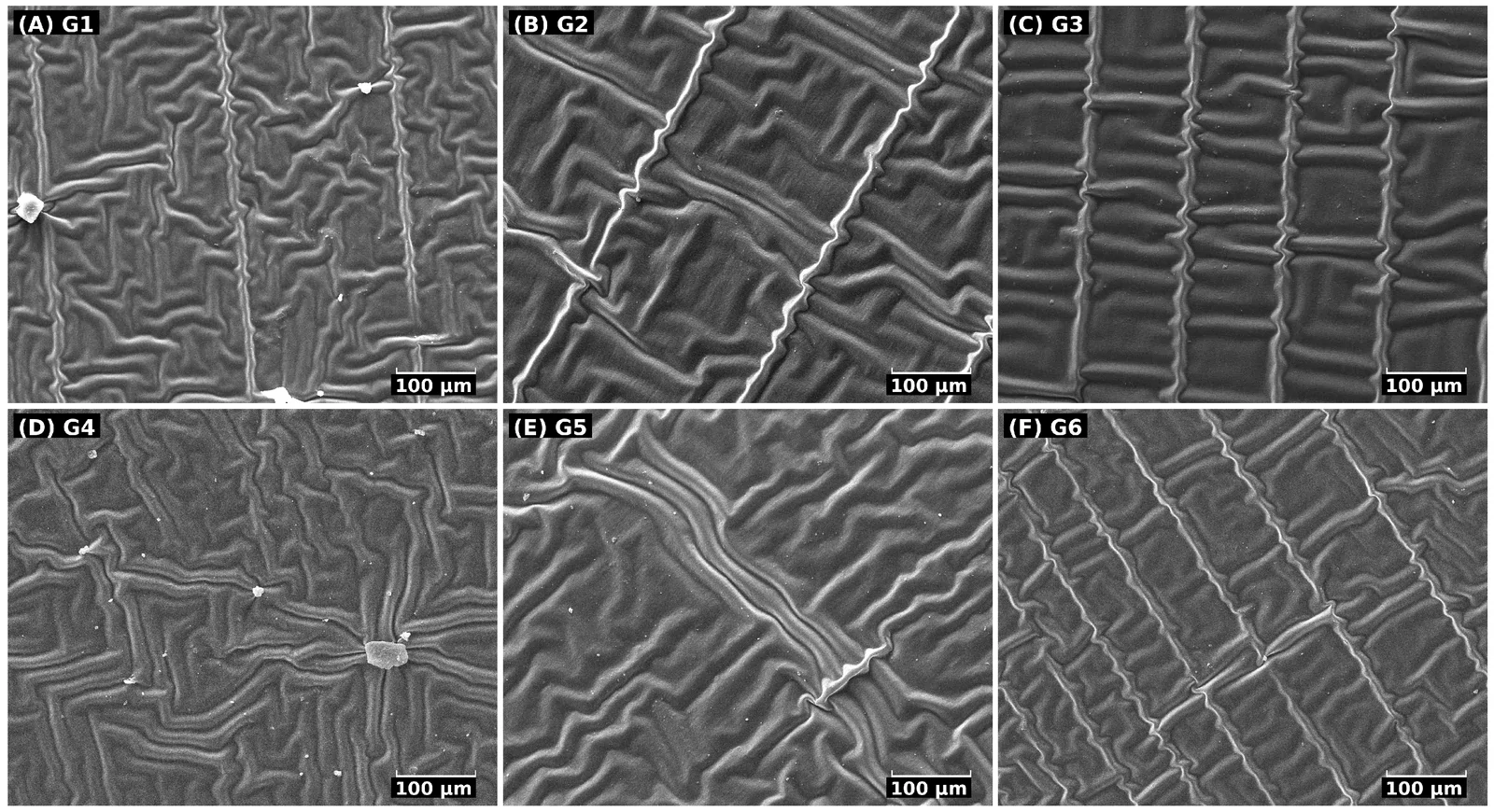
Representative post-fracture FESEM micrographs of directly printed aligner specimens at 1000× magnification according to printing layer thickness and thermocycling condition: (**A**) G1, 50 µm non-thermocycled; (**B**) G2, 50 µm thermocycled; (**C**) G3, 100 µm non-thermocycled; (**D**) G4, 100 µm thermocycled; (**E**) G5, 150 µm non-thermocycled and (**F**) G6, 150 µm thermocycled.

Representative FESEM micrographs of the pre-fracture surface morphology and post-fracture cross-sections of the directly printed aligner specimens are shown in Figure 4 and Figure 5, respectively. The FESEM observations demonstrated clear morphological differences according to printing layer thickness and thermocycling condition.

#### 3.2.1. Pre-Fracture FESEM

The 50 µm specimens, before thermocycling, showed the most uniform and continuous surface morphology, with closely spaced parallel striations corresponding to the layer-by-layer printing process and minimal surface irregularities (Figure 4A). No obvious cracks, voids, or interlayer separation were observed. After thermocycling, the 50 µm specimens maintained general surface continuity; however, increased surface irregularity, granular deposits, localized fissures, and partial masking of the layered architecture were observed (Figure 4B).

The 100 µm specimens before thermocycling showed a relatively organized surface with visible interlayer boundaries, regular ridge-and-valley patterns, and moderate surface irregularity (Figure 4C). Minor localized irregularities and scattered particulate deposits were observed, but the overall surface continuity was preserved. Following thermocycling, the 100 µm specimens exhibited increased surface irregularity, less distinct interlayer boundaries, localized discontinuities, and a greater number of microcrack-like and void-like defects along the interlayer regions (Figure 4D).

The 150 µm specimens demonstrated more pronounced layer-related surface features compared with the 50 µm and 100 µm groups. Before thermocycling, the 150 µm specimens showed distinct interlayer boundaries, increased surface heterogeneity, micro-voids, irregular cavities, and occasional microcrack-like features (Figure 4E). After thermocycling, the 150 µm specimens exhibited the most evident surface alterations among all groups, including pronounced ridges and valleys, increased surface irregularity, numerous microcrack-like features, void-like defects, particulate structures, and disrupted layer continuity (Figure 4F).

#### 3.2.2. Post-Fracture FESEM

Post-fracture FESEM analysis further demonstrated differences in internal morphology among the tested groups (Figure 5). The 50 µm specimens before thermocycling showed a compact and continuous fracture surface, with well-organized printed layers and no evidence of gross delamination (Figure 5A). After thermocycling, the fracture surface of the 50 µm specimens became more irregular, with fine discontinuities, micro-voids, localized nodular features, and slight distortion of interlayer boundaries (Figure 5B).

The 100 µm specimens showed a well-defined layered fracture morphology before thermocycling, with fine cracks, voids, and localized surface heterogeneity (Figure 5C). After thermocycling, the fracture surface exhibited more prominent interlayer irregularity, micro-gaps, discontinuities, and crack-like discontinuities occurring predominantly along the layer interfaces (Figure 5D).

The 150 µm specimens showed the most distinct layer-wise fracture pattern. Before thermocycling, prominent interlayer boundaries, stepwise morphology, small defects, and interlayer void-like features were observed (Figure 5E). After thermocycling, the 150 µm specimens showed the greatest degree of fracture surface disruption, with increased occurrence of microcrack-like features, reduced surface uniformity, more visible interlayer interfaces, localized defects, and discontinuities within the printed structure (Figure 5F).

Overall, representative FESEM micrographs revealed qualitative differences in surface and fracture morphology according to printing layer thickness and thermocycling condition. Greater layer thickness was associated with more visible layer boundaries and greater surface heterogeneity. Post-thermocycling specimens showed more discontinuities, cracks, and void-like features, with the most evident qualitative alterations observed in the 150 µm group. Because the observations were based on representative micrographs, they were not quantified or statistically compared.

### 3.3. Light Transmittance Measurement

**Figure 6:**
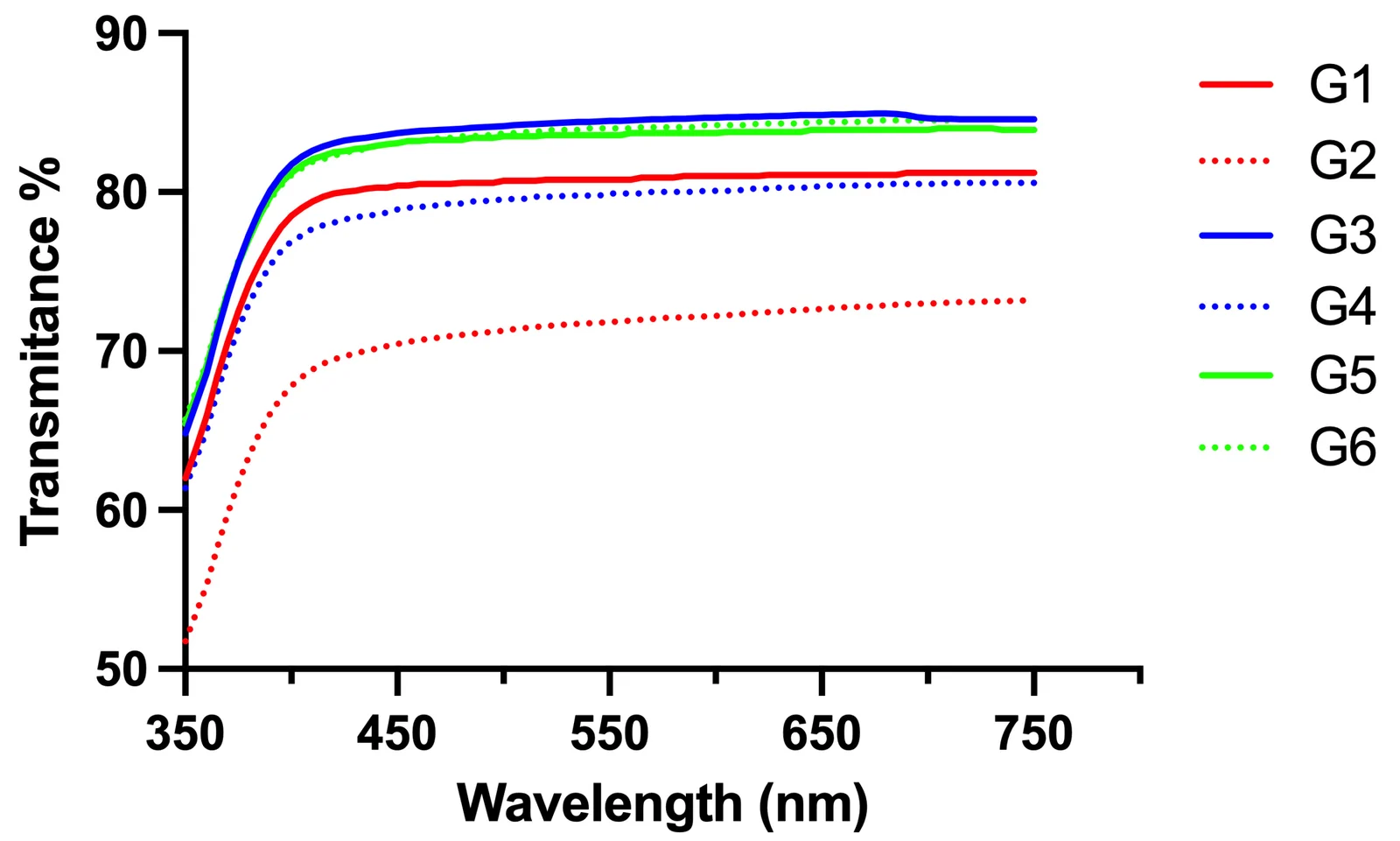
Mean spectral transmittance (%) of directly printed aligner specimens with different layer thicknesses (50, 100, and 150 µm) before and after thermocycling across the wavelength range of 350–750 nm. G1–G6 represent the experimental groups.

For the three printing layer thickness groups (50, 100, and 150 µm), the transmittance before thermocycling treatment was quite similar throughout the measured spectral range of 350–750 nm (Figure 6). After thermocycling, the 50 µm group showed a marked decrease in transmittance, while the 100 µm group showed a decrease in transmittance that was not as marked, and the 150 µm group showed practically no variation (Figure 6).

This was confirmed by the quantification of the AUC of the transmittance spectra (Table 8). The differences between groups were statistically significant (*p* < 0.001). The thermocycling × layer-thickness interaction was significant (*p* = 0.001), indicating that the effect of thermocycling on normalized light transmittance depended on printing layer thickness.

**Figure 7:**
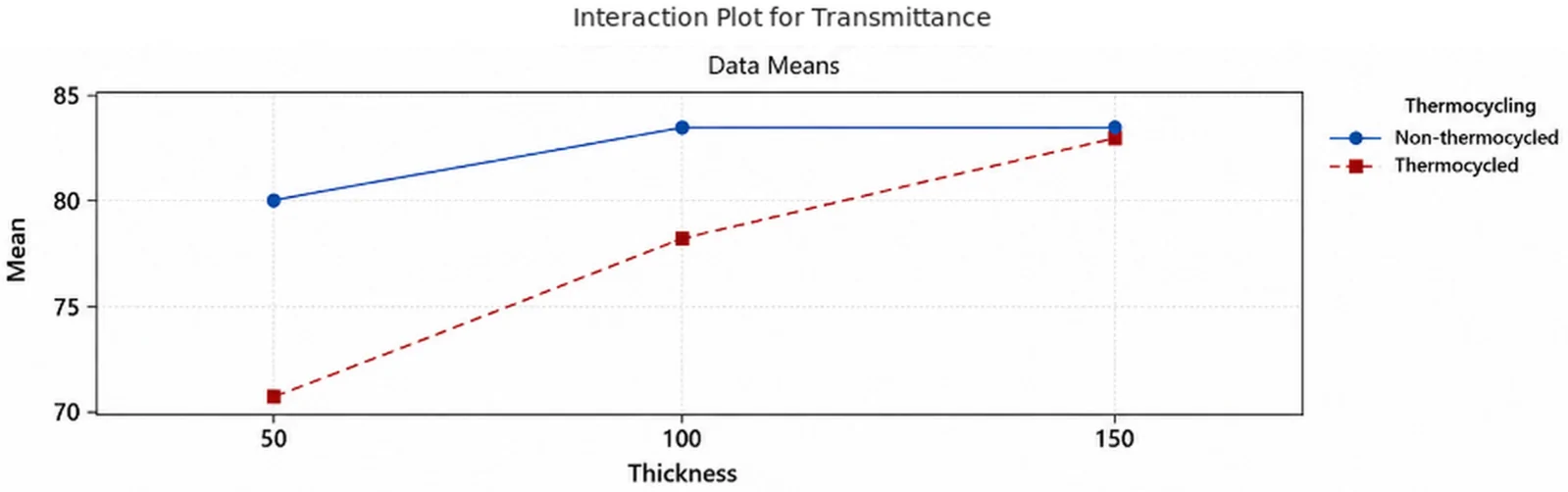
Interaction effect of layer thickness and thermocycling on transparency.

The interaction plot (Figure 7) showed that the 50 and 150 µm thickness groups had the largest and smallest decrease in transparency, respectively; this further supports the statistical interaction.

### 3.4. Dimensional Accuracy

#### 3.4.1. Trueness

Trueness was evaluated by comparing the intaglio surface of each scanned aligner with the reference STL file. Lower RMS deviation indicated better trueness. Descriptive statistics and Tukey groupings are presented in Table 9.

The transformed trueness response satisfied the assumptions of normality and homogeneity of variance. Two-way ANOVA showed a significant effect of thermocycling (*p* = 0.006) and a significant thermocycling × layer-thickness workflow interaction (*p* = 0.008), whereas layer-thickness workflow alone was not significant (*p* = 0.887). The interaction was, therefore, interpreted jointly.

**Figure 8:**
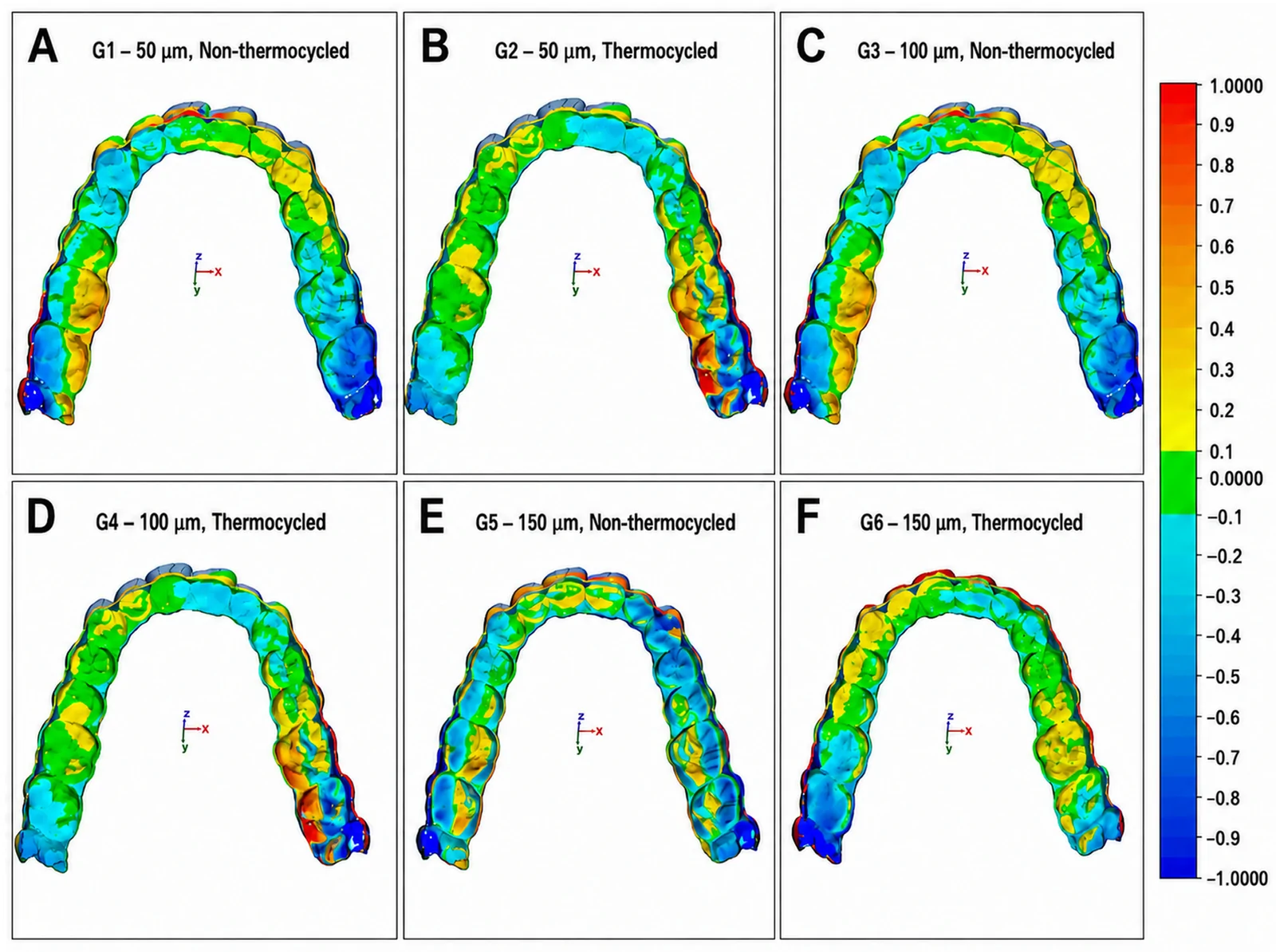
Representative three-dimensional color maps showing the trueness of directly printed aligners according to layer-thickness workflow and thermocycling condition: (**A**) G1, 50 µm non-thermocycled; (**B**) G2, 50 µm thermocycled; (**C**) G3, 100 µm non-thermocycled; (**D**) G4, 100 µm thermocycled; (**E**) G5, 150 µm non-thermocycled and (**F**) G6, 150 µm thermocycled. The tolerance was set at ±0.10 mm, with a common displayed deviation range of −1.00 to +1.00 mm. Lower RMS deviation indicates greater trueness.

Before thermocycling, the 150 µm workflow showed the lowest mean RMS deviation, while the 50 µm workflow showed the highest and most variable deviation (Table 9). Thermocycling produced a workflow-dependent response: mean deviation decreased numerically in the 50 µm workflow but increased in the 100 and 150 µm workflows. The greatest mean deviation after thermocycling occurred in the 150 µm workflow. Representative three-dimensional deviation maps illustrating the trueness patterns across the six experimental conditions are shown in Figure 8.

Tukey-adjusted comparisons showed that the thermocycled 100 and 150 µm groups had significantly greater RMS deviations than the non-thermocycled 150 µm group (*p* < 0.05); no other pairwise comparisons were significant. These findings indicate that dimensional trueness after thermocycling depended on the printing workflow rather than on layer thickness alone.

#### 3.4.2. Precision

The transformed precision response satisfied the assumptions of normality and homogeneity of variance. Two-way ANOVA showed significant effects of thermocycling (*p* < 0.001), printing layer-thickness workflow (*p* < 0.001), and their interaction (*p* = 0.034). Because the interaction was significant, the effects of thermocycling and layer-thickness workflow were interpreted jointly.

Before thermocycling, the three workflows showed similar mean RMS deviations, although the 50 µm workflow showed greater variability (Table 10). Thermocycling produced a workflow-dependent response: mean deviation decreased slightly in the 50 µm workflow but increased in the 100 and 150 µm workflows. The thermocycled 100 µm workflow showed the greatest mean RMS deviation and, therefore, the lowest precision.

Tukey-adjusted comparisons showed that the thermocycled 100 µm group had significantly greater RMS deviations than all other groups (*p* < 0.05). The thermocycled 150 µm group also showed significantly greater deviation than the non-thermocycled 50 µm group (*p* < 0.05); no other pairwise comparisons were significant. These findings indicate that manufacturing precision after thermocycling depended on the printing workflow, with the greatest deterioration observed in the thermocycled 100 µm group.

## 4. Discussion

The present study evaluated the effects of printing layer-thickness workflow and short-term thermocycling on the mechanical, morphological, optical, and dimensional properties of a directly printable orthodontic polymer. The null hypotheses were rejected for several outcomes because printing layer thickness, thermocycling, or their interaction significantly affected the evaluated properties. Under the standardized centrifugation and nitrogen post-curing conditions, the 50 µm workflow provided the most favorable combination of strain at break and flexural force, strength, and modulus. Thermocycling reduced Young’s modulus and all evaluated flexural outcomes, although the magnitude and statistical pattern of the response varied among properties. Stress at peak, peak deflection, microhardness, and normalized light transmittance demonstrated significant interactions, indicating that their responses to thermocycling depended on the selected layer-thickness workflow. In contrast, the interaction was not significant for strain at break. For this outcome, printing layer thickness was significant, whereas thermocycling and the thermocycling × printing layer thickness interaction were not significant. Collectively, these findings demonstrate that the performance of directly printed aligner polymers is governed by a process–structure–property relationship and that no single manufacturing setting necessarily optimizes all clinically relevant characteristics.

The 50 µm specimens exhibited the highest strain-at-break values and significantly superior flexural properties compared with the 100 and 150 µm specimens. For stress at peak, neither the main effect of thermocycling nor that of printing layer thickness was statistically significant. However, the printing layer thickness × thermocycling interaction was significant, indicating that the effect of thermocycling differed according to the selected layer-thickness workflow. Numerically, stress at peak increased after thermocycling in the 50 µm group but decreased in the 100 and 150 µm groups. For strain at break, printing layer thickness had a significant overall effect, whereas thermocycling and the printing layer thickness × thermocycling interaction were not significant. The 50 µm group exhibited the highest strain-at-break values under both thermocycling conditions. Although thermocycling numerically increased strain at break in the 50 µm group, produced no change in the mean of the 100 µm group, and decreased it in the 150 µm group, these group-specific changes should be regarded as descriptive because the interaction was not statistically significant. These findings suggest that the finer-layer workflow may have produced a more mechanically favorable architecture for TA-28 under the investigated manufacturing conditions, particularly in relation to strain at break and flexural performance. Overall, the lack of significant differences in the primary flexural metrics between the 100 and 150 µm conditions suggests that the relationship was not strictly linear across the tested layer thicknesses. It is possible that thinner layers produced greater material continuity and reduced the mechanical consequences of layer boundaries. This mechanism was not quantitatively demonstrated; however, FESEM examination of non-thermocycled 50 µm specimens qualitatively showed a more uniform surface and more compact fracture morphology than specimens produced with thicker layers, which exhibited more apparent layer boundaries, heterogeneity, and void-like features. Similarly, Farkas et al. reported that layer thickness and orientation affected the tensile properties of a DLP dental resin, with higher tensile values at 50 µm than at 100 µm [5]. However, because resin chemistry, orientation, exposure, and post-curing conditions differ among studies, these findings support a guideline specific to TA-28 and the investigated workflow rather than a universal 50 µm rule [5,6,14]. Consistent with this workflow-dependent interpretation, Wu et al. found that printing layer thickness and build orientation significantly affected the mechanical properties, surface roughness, and color stability of directly printed clear-aligner resin [8]. Recent direct-aligner evidence further indicates that printer type and resin selection can substantially influence mechanical behavior, reinforcing that the present findings should be interpreted as workflow-specific [8,23].

The restricted factorial design was selected to answer one focused question: how do three layer-thickness-specific TA-28 workflows respond to short-term thermocycling under a standardized printer, orientation, washing, post-curing, and final thermal conditioning protocol? This focus does not imply that exposure time and build orientation are unimportant. Exposure time affects the delivered energy per layer and can change polymer conversion and thermomechanical behavior [7]. Build orientation can alter the direction of applied load relative to layer interfaces; direct-aligner evidence is mixed, with one study reporting orientation-dependent mechanical and surface outcomes [8] and another finding no horizontal-versus-vertical difference [9]. These parameters were held constant rather than factorially varied because testing them jointly with three layer thicknesses and two aging conditions would require a substantially larger design.

The 150 µm condition nevertheless required adjustments to exposure and movement parameters, whereas the 50 and 100 µm conditions used manufacturer-recommended settings. Consequently, differences involving the 150 µm group cannot be attributed exclusively to geometric layer thickness and should be interpreted as the combined effect of a layer-thickness-specific manufacturing workflow. This limitation underscores the importance of reporting slicing, exposure, movement, support, washing, build orientation, and post-curing parameters when interpreting process–property relationships [20,24]. Post-processing should not be considered a technical afterthought, but rather an integral part of the manufacturing workflow. In a study of TC-85 polyurethane aligners, rinsing protocol and polymerization environment affected flexural modulus and hardness, with centrifugation followed by nitrogen polymerization producing the highest flexural modulus [14]. A review of 3D-printed dental appliances likewise identified support removal, washing, secondary polymerization, and surface treatment as important stages affecting mechanical, biological, dimensional, and esthetic properties [24].

Young’s modulus, force at peak, flexural strength, flexural modulus, and deflection at peak decreased after thermocycling. The 150 µm group exhibited the greatest numerical decrease in deflection at peak. Because the printing layer thickness × thermocycling interaction was significant for deflection at peak (*p* = 0.010), the effect of thermocycling on this outcome depended on the selected layer-thickness workflow. In contrast, the absence of significant interactions for flexural force, flexural strength, and flexural modulus suggests that the reduction associated with thermocycling was generally comparable across the tested layer-thickness workflows. The reductions in flexural properties were qualitatively consistent with the increased interfacial irregularities, microcrack-like features, fissure-like discontinuities and void-like features observed by FESEM. Nevertheless, the qualitative micrographs cannot establish a direct causal relationship between these morphological features and the measured mechanical changes. The thermal challenge used in this study alternated specimens between 5 and 55 °C in water. Repeated temperature changes may impose cyclic thermal strain, while water exposure may promote plasticization, stress relaxation, or changes at poorly integrated interlayer regions. These mechanisms are consistent with the reductions in Young’s modulus and flexural outcomes observed after thermocycling, but they were not measured directly and, therefore, remain mechanistic hypotheses rather than demonstrated chemical changes. A 7-day clinical retrieval study of another Graphy resin (TC-85 DAC) reported significant changes in elastic index and indentation relaxation in directly printed aligners relative to unused specimens [25]. The findings are not directly comparable with the present results because the resin formulation, aging exposure, specimen geometry, testing methods, and mechanical endpoints differed.

The microhardness findings indicated that the effect of aging depended on layer thickness: a significant interaction between layer thickness and thermocycling was observed, whereas thermocycling alone was not significant. The increase in hardness at 50 µm and the decrease at 100 and 150 µm cannot, therefore, be interpreted as general thermocycling-induced hardening or softening of TA-28. Rather, the local surface response may depend on printed architecture and effective curing history. This interpretation is consistent with orthodontic polymer evidence showing that thermocycling effects vary according to material and endpoint. Albilali et al. reported that thermocycling generally increased flexural modulus and hardness across six thermoformed retainer materials, while significant surface-roughness increases occurred in only two materials [26]. However, microhardness is not a direct measure of clinical abrasion resistance, biofilm retention, or fatigue. These outcomes require dedicated wear, fatigue, quantitative roughness, and biological-adhesion testing protocols [5,26].

The statistically significant interactions are consistent with, but do not prove, a process–structure–property explanation. A thinner-layer workflow may create more closely spaced interfaces and a different local curing history; after thermal–aqueous cycling, water uptake and repeated thermal strain could affect bulk polymer mobility and interfacial regions differently across those architectures. The qualitative FESEM observations—more conspicuous layer boundaries and void-like and fissure-like discontinuities in the thicker-layer groups, particularly after thermocycling—are compatible with this interpretation. However, FESEM images from representative specimens cannot quantify porosity, interfacial bond strength, roughness, or defect density, and they cannot establish that the observed features caused the mechanical, hardness, or optical interactions. The current data, therefore, support a structural hypothesis, not a demonstrated physicochemical mechanism. Direct testing would require, at minimum, degree-of-conversion measurements (e.g., ATR-FTIR or Raman), water sorption/solubility, quantitative roughness or profilometry, and fracture or fatigue testing; such measurements would distinguish altered conversion, water plasticization, and interfacial damage as competing explanations.

Normalized light transmittance was an important secondary outcome because optical appearance is relevant to the esthetic performance of clear aligners. Baseline transmittance values were numerically similar among groups. However, thermocycling produced the greatest reduction in the 50 µm group, a smaller reduction in the 100 µm group, and only minimal change in the 150 µm group. Thus, the workflow with the most favorable overall mechanical profile also showed the greatest thermocycling-associated reduction in normalized light transmittance. The functional or perceptual significance of these changes was not assessed in the present study. This finding should not be interpreted as evidence of color stability because color difference and staining were not measured. In a study of printed dental resins, post-printing conditions affected flexural strength and wear, whereas hydrothermal aging reduced flexural strength; optical properties did not significantly differ across the tested post-printing conditions [27]. The difference from the present findings may reflect differences in resin formulation, post-processing protocol, aging exposure, and optical endpoint [27].

The thermocycling-associated differences in the 350–750 nm transmittance curves, including the region from approximately 650 to 750 nm, describe changes in visible-light transmission through the specimens. Because this measurement was not a vibrational spectroscopic analysis, it does not identify specific functional groups, bond vibrations, or chemical reactions. The observed spectral differences may reflect changes in light scattering or absorption associated with surface/interfacial irregularities, water-related changes, or other physical alterations; they must not be interpreted as direct evidence of molecular-bond rearrangement.

The FESEM analysis adds a morphological dimension to the mechanical and optical findings. With increasing layer thickness, FESEM images showed more defined layer boundaries and greater surface heterogeneity. In thermocycled conditions, more frequent crack-like and fissure-like discontinuities and void-like features were observed, particularly in the 150 µm group. These findings underscore the importance of considering printing layer thickness and thermocycling when optimizing directly printed aligners. However, FESEM evaluation, particularly when based on representative micrographs, remains qualitative and cannot quantify defect density, surface irregularity, or interfacial bond strength. Future studies should, therefore, combine FESEM observations with quantitative surface characterization, such as profilometry or atomic force microscopy, and additional analyses of interfacial integrity, water sorption and solubility, degree of conversion, fatigue, and wear after combined thermal, chemical, and staining challenges.

The dimensional-accuracy results demonstrated significant thermocycling × layer-thickness workflow interactions for both trueness and precision, indicating that thermocycling did not produce a uniform dimensional response across the three printing workflows. The 50 µm workflow showed a numerical reduction in RMS deviation after thermocycling, whereas the 100 and 150 µm workflows showed increased deviations. These findings support the interpretation that dimensional stability in directly printed aligners depends on the overall manufacturing workflow rather than nominal layer thickness alone. Differences in layer architecture, interlayer continuity, curing history, polymer-chain mobility, water-related plasticization, and internal residual stresses may contribute to these workflow-dependent responses. However, these mechanisms were not directly evaluated in the present study and should, therefore, be considered hypothetical.

Dimensional stability may also be influenced by post-processing conditions. Avolese et al. [28] demonstrated that removal of printing supports before final curing affected dimensional accuracy in directly printed aligners. Dantagnan et al. [4] similarly reported that exposure to artificial saliva affected the trueness and precision of printed and thermoformed aligners. These aging-related findings are consistent with the increased RMS deviations observed for the thermocycled 100 and 150 µm workflows in the present study, but they do not explain the numerical reduction observed for the 50 µm workflow. Differences in resin composition, printing and post-processing procedures, aging protocols, and three-dimensional alignment methods may contribute to these contrasting responses.

Since no thermoformed comparator was included in the present study, it cannot be determined whether the TA-28 workflows produced greater or lower dimensional trueness and precision than conventionally manufactured aligners. In an in vitro study, Koenig et al. [3] reported greater trueness and precision for a directly 3D-printed aligner material than for two thermoformed aligner materials; however, those findings may not be applicable to TA-28 or the manufacturing workflows evaluated in the present study. Therefore, the present results should be interpreted as evidence of workflow-dependent dimensional stability following thermocycling and should not be considered evidence of clinical superiority or differences in orthodontic force delivery between manufacturing systems.

Several limitations should be considered when interpreting the present findings. Only one directly printable resin, one LCD-based stereolithography printer, one fixed build orientation for each specimen type (0° for flat test specimens and 45° for full-arch aligners), and one post-processing protocol were evaluated. Exposure time and build orientation were not independently varied; therefore, their main effects and interactions with layer thickness and thermocycling cannot be estimated from this experiment, and the findings should not be generalized to other resin–printer combinations or manufacturing workflows. Furthermore, because the 150 µm workflow required adjustments to the exposure and movement parameters, differences involving this group represent the combined effect of the layer-thickness-specific manufacturing workflow rather than geometric layer thickness alone. The standardized specimens and 250 thermocycling cycles represented a controlled short-term thermal challenge and did not reproduce the complex geometry of complete aligners or the combined effects of occlusal loading, mechanical fatigue, saliva, pH fluctuations, dietary staining, biofilm accumulation, and long-term clinical wear. In addition, FESEM observations were qualitative and based on representative specimens; therefore, they could not quantify defect density or establish a causal relationship between the observed crack-like, fissure-like, and void-like morphological features and mechanical performance. The study also did not determine whether the measured mechanical and optical differences exceeded clinically perceptible or acceptable thresholds. Nevertheless, the factorial design demonstrated that TA-28 performance depended on the selected manufacturing workflow. The 50 µm workflow provided the highest strain at break and significantly superior flexural properties, whereas the 150 µm workflow showed more stable normalized light transmittance after thermocycling but lower flexural performance and more pronounced qualitative morphological irregularities. Future studies should evaluate complete aligners under combined thermal and mechanical aging and investigate force delivery, fit, color stability, wear, comfort, and biological safety throughout the clinical service period [22]. Additional water-aging and water-sorption studies are also warranted because aqueous exposure can alter the mechanical behavior of printed aligner materials [29].

Chemical characterization was not performed. Consequently, the study cannot determine whether thermocycling altered the degree of conversion, molecular bonds, surface chemistry, or chemical composition of TA-28. Future work should combine transmittance measurements with FTIR or Raman spectroscopy to assess functional-group and degree-of-conversion changes; XPS may be used to examine surface elemental chemistry, while fluorescence methods may help characterize optical aging or residual photoinitiator-related changes.

From a practical manufacturing perspective, the present results show that the printing workflow for directly printed aligners should not be selected on production speed alone. Under the standardized conditions used here, the 50 µm workflow retained the most favorable mechanical profile and comparatively favorable dimensional behavior in thermocycled conditions, but it also showed the greatest decrease in normalized light transmittance. Measurement of light transmittance before and after controlled thermal aging may, therefore, provide a simple, non-destructive complementary approach for screening optical aging of printed aligner materials. However, it should be interpreted with mechanical testing, dimensional analysis, surface characterization, and chemical spectroscopy, rather than as a stand-alone indicator of polymer degradation or molecular change.

## 5. Conclusions

Within the limitations of this in vitro study, printing layer-thickness workflow and thermocycling affected the mechanical, morphological, optical, and dimensional properties of directly printed Graphy Tera Harz TA-28 aligner material. The 50 µm workflow provided the most favorable overall mechanical performance, with greater strain at break and superior flexural properties than the 100 and 150 µm workflows. Thermocycling reduced Young’s modulus and the measured flexural outcomes, whereas the effects on peak tensile stress, microhardness, peak deflection, normalized light transmittance, trueness, and precision depended on the selected workflow. Dimensional accuracy showed significant workflow-dependent responses to thermocycling: the 100 and 150 µm workflows showed increased dimensional deviation after thermocycling, whereas the 50 µm workflow showed a numerical reduction in RMS deviation. Overall, the 50 µm workflow provided the most favorable mechanical profile and maintained comparatively favorable dimensional behavior after thermocycling, although it showed the greatest reduction in normalized light transmittance. These findings do not establish clinical fit, force delivery, or superiority over thermoformed aligners because the study did not include a thermoformed comparator. Thus, the selection of the layer-thickness workflow involves a balance among mechanical performance, dimensional stability, and optical behavior. This conclusion applies to the standardized manufacturing and post-processing protocol used in this particular study and does not establish an optimal post-processing condition.

## Figures and Tables

**Table 1:** Experimental groups according to printing layer thickness and thermocycling condition.

Group	Printing Layer Thickness	Thermocycling Condition
G1	50 µm	Non-thermocycled
G2	50 µm	Thermocycled
G3	100 µm	Non-thermocycled
G4	100 µm	Thermocycled
G5	150 µm	Non-thermocycled
G6	150 µm	Thermocycled

**Table 2:** Descriptive statistics (mean ± standard deviation) of the tensile properties according to printing layer thickness and thermocycling condition (*n* = 5 per group).

Thickness (µm)	Thermocycling	Stress at Peak (MPa)	Strain at Break (%)	Young’s Modulus (MPa)
50	Non-thermocycled	35 ± 5.10	153.6 ± 29.3	479.6 ± 39.72
	Thermocycled	38.6 ± 6.11	183.6 ± 40.83	415.6 ± 57.91
100	Non-thermocycled	43.2 ± 4.09	108.2 ± 32.28	517.2 ± 49.37
	Thermocycled	37.00 ± 2.55	108.2 ± 42.96	445.4 ± 47.80
150	Non-thermocycled	39.00 ± 2.12	123.2 ± 44.82	486.6 ± 8.35
	Thermocycled	34.80 ± 3.27	85.2 ± 53.69	458.6 ± 12.20

**Table 3:** Two-way ANOVA results for the effects of thermocycling, printing layer thickness, and their interaction on the tensile properties.

Property	Source	*p*-Value
Stress at Peak (MPa)	Thermocycling	0.159
	Thickness	0.179
	Thermocycling × Thickness	0.035
Strain at Break (%)	Thermocycling	0.858
	Thickness	0.003
	Thermocycling × Thickness	0.204
Young’s Modulus (MPa)	Thermocycling	0.001
	Thickness	0.172
	Thermocycling × Thickness	0.451

**Table 4:** Descriptive statistics (mean ± standard deviation) of the flexural properties of directly printed clear-aligner specimens according to printing layer thickness and thermocycling condition (*n* = 5 per group).

Thickness (µm)	Thermocycling	Force at Peak (N) Mean ± SD	Deflection at Peak (mm) Mean ± SD	Flexural Strength (MPa) Mean ± SD	Flexural Modulus (MPa) Mean ± SD
50	Non-thermocycled	91.46 ± 5.68	7.21 ± 0.39	62.99 ± 3.91	1565.9 ± 134.11
	Thermocycled	78.2 ± 3.17	7.15 ± 0.19	53.86 ± 2.19	1413.16 ± 62.04
100	Non-thermocycled	83.5 ± 3.68	6.98 ± 0.29	57.51 ± 2.54	1440.93 ± 32.74
	Thermocycled	66.74 ± 4.11	6.83 ± 0.44	45.96 ± 2.83	1202.26 ± 62.41
150	Non-thermocycled	83.18 ± 2.93	7.75 ± 0.56	57.29 ± 2.02	1383.41 ± 79.02
	Thermocycled	70.22 ± 6.5	6.64 ± 0.38	48.36 ± 4.47	1281.68 ± 116.93

**Table 5:** Two-way analysis of variance (ANOVA) results for the effects of thermocycling, printing layer thickness, and their interaction on the flexural properties of directly printed aligner specimens.

Response Variable	Source	*p*-Value
Force at Peak (N)	Thermocycling	<0.001
	Thickness	<0.001
	Thermocycling × Thickness	0.589
Deflection at Peak (mm)	Thermocycling	0.005
	Thickness	0.193
	Thermocycling × Thickness	0.010
Bending Strength (MPa)	Thermocycling	<0.001
	Thickness	<0.001
	Thermocycling × Thickness	0.589
Bending Modulus (MPa)	Thermocycling	<0.001
	Thickness	<0.001
	Thermocycling × Thickness	0.235

**Table 6:** Descriptive statistics of Vickers microhardness according to the printing layer thickness and thermocycling (*n* = 5 per group).

Thickness (µm)	Thermocycling	Vickers Microhardness, HV Mean ± SD
50	Non-thermocycled	10.13 ± 0.60
	Thermocycled	10.36 ± 0.26
100	Non-thermocycled	10.34 ± 1.00
	Thermocycled	8.79 ± 0.61
150	Non-thermocycled	11.36 ± 2.30
	Thermocycled	10.62 ± 2.08

**Table 7:** Two-way analysis of variance (ANOVA) investigating Vickers microhardness differences following Box-Cox transformation.

Source	*p*-Value
Thickness	0.026
Thermocycling	0.055
Thickness × Thermocycling	0.037

Note: Vickers microhardness data were Box–Cox transformed using the optimal λ. Descriptive statistics are reported in HV, whereas *p*-values are based on the transformed data.

**Table 8:** Normalized light transmittance values (mean ± standard deviation) of directly printed aligner specimens fabricated at different layer thicknesses of non-thermocycled and thermocycled (*n* = 5 per group).

Layer Thickness (µm)	Thermocycling	Normalized Light Transmittance (Mean ± SD)
50	Non-thermocycled	79.90 ± 1.05
	Thermocycled	71.08 ± 2.69
100	Non-thermocycled	83.00 ± 1.09
	Thermocycled	78.23 ± 3.58
150	Non-thermocycled	82.78 ± 2.39
	Thermocycled	82.56 ± 0.99

Two-way ANOVA: layer thickness, *p* < 0.001; thermocycling, *p* < 0.001; layer thickness × thermocycling interaction, *p* = 0.001.

**Table 9:** Trueness of directly printed aligners according to layer-thickness workflow and thermocycling condition.

Layer-Thickness Workflow	Non-Thermocycled RMS (mm), Mean ± SD	Tukey Group	Thermocycled RMS (mm), Mean ± SD	Tukey Group
50 µm	0.62 ± 0.28	AB	0.50 ± 0.09	AB
100 µm	0.45 ± 0.04	AB	0.69 ± 0.31	A
150 µm	0.40 ± 0.10	B	0.81 ± 0.34	A

Note: Five aligners were evaluated per condition. Descriptive values are presented on the original scale. Tukey grouping letters were generated from the transformed trueness response; means without a shared letter differed at *p* < 0.05. Lower RMS deviation indicates greater trueness.

**Table 10:** Precision of directly printed aligners according to layer-thickness workflow and thermocycling condition.

Layer-Thickness Workflow	Non-Thermocycled RMS (mm), Mean ± SD	Tukey Group	Thermocycled RMS (mm), Mean ± SD	Tukey Group
50 µm	0.50 ± 0.36	C	0.44 ± 0.13	BC
100 µm	0.47 ± 0.14	BC	0.81 ± 0.23	A
150 µm	0.47 ± 0.07	BC	0.57 ± 0.10	B

Note: Five aligners were evaluated per condition. Descriptive values are presented on the original scale. Tukey grouping letters were generated from the transformed precision response; means without a shared letter differed at *p* < 0.05. Lower RMS deviation indicates greater precision.

## Data Availability

The data presented in this study are available on request from the corresponding author.

## Abbreviations

The following abbreviations are used in this manuscript:

CAT	Clear Aligner Therapy
FESEM	Field-Emission Scanning Electron Microscopy
STL	Standard Tessellation Language
CAD	Computer-Aided Design
LED	Light-Emitting Diode
AUC	Area Under Curve
ANOVA	Analysis of Variance
RMS	Root Mean Square

## Appendix A

The printing parameters used for each layer thickness are presented in Table A1. The 50 and 100 µm settings were based on the manufacturer-recommended profiles, whereas the 150 µm profile was developed by systematically adjusting the 100 µm parameters. The normal exposure time was increased in increments of 0.3 s, from 0.8 s at 50 µm to 1.1 s at 100 µm and subsequently to 1.4 s at 150 µm. The initial exposure time was increased from 5 s at 50 µm to 7 s at 100 µm and to 8 s at 150 µm. The rise height for the 150 µm workflow was maintained at 8 mm, consistent with the manufacturer-recommended setting for the 100 µm workflow.

The number of initial layers was reduced as the layer thickness increased. Six initial layers were used at 50 µm and three at 100 µm. Following the same proportional reduction, dividing three layers by two resulted in a theoretical value of 1.5 layers for the 150 µm workflow; because a fractional layer cannot be applied, this value was rounded to two layers. This selection also maintained an approximately constant total initial-layer thickness of 300 µm across the three workflows: 6 × 50 µm, 3 × 100 µm, and 2 × 150 µm. Motor speed, LED power, initial LED power, and the number of transition layers were kept constant to minimize variation between the printing workflows.

**Table A1:** Printing parameters used for each printing-layer thickness.

Parameter	50 µm (Manufacturer)	100 µm (Manufacturer)	150 µm (Developed)
Exposure Time	0.8 s	1.1 s	1.4 s
Initial Exposure	5.0 s	7 s	8 s
Motor Speed	800 mm/min	800 mm/min	800 mm/min
Rise Height	6 mm	8 mm	8 mm
Rise Height (Initial)	10 mm	12 mm	12 mm
LED power	85%	85%	85%
LED power (Initial)	100%	100%	100%
Initial Layers	6	3	2
Transition Layers	7	7	7
Test-specimen build orientation	0°	0°	0°
Test-specimen supports	None	None	None
Aligner build orientation	45°	45°	45°
Aligner support configuration	Distributed supports; reduced density on labial surface	Distributed supports; reduced density on labial surface	Distributed supports; reduced density on labial surface
